# Semantic Communication Based on Slot Attention for MIMO Transmission in 6G Smart Factories

**DOI:** 10.3390/s26082456

**Published:** 2026-04-16

**Authors:** Na Chen, Guijie Lin, Rubing Jian, Yusheng Wang, Meixia Fu, Jianquan Wang, Lei Sun, Wei Li, Taisei Urakami, Minoru Okada, Bin Shen, Qu Wang, Changyuan Yu, Fangping Chen, Xuekui Shangguan

**Affiliations:** 1 School of Automation and Electrical Engineering, University of Science and Technology Beijing, Beijing 100083, China; chenna@ustb.edu.cn (N.C.); d202510386@xs.ustb.edu.cn (G.L.); m202410532@xs.ustb.edu.cn (Y.W.); mxfu1205@ustb.edu.cn (M.F.); wangqu@ustb.edu.cn (Q.W.); 2 Beijing Key Laboratory of Industrial Deterministic Networking and Intelligent Collaborative Control, University of Science and Technology Beijing, Beijing 100083, China; m202420971@xs.ustb.edu.cn (R.J.); li_wei@ustb.edu.cn (W.L.); 3 School of Computer and Communication Engineering, University of Science and Technology Beijing, Beijing 100083, China; 4 National Institute of Technology, Kagawa College, Takamatsu 760-8521, Kagawa, Japan; urakami-t@cn.kagawa-nct.ac.jp; 5 Information Science Division, Graduate School of Science and Technology, Nara Institute of Science and Technology, Ikoma 630-0192, Nara, Japan; mokada@is.naist.jp; 6 Key Laboratory of Industrial Internet Network Innovation and Test Verification, Ministry of Industry and Information Technology, China Academy of Information and Communications Technology, Beijing 100191, China; 7 Shunde Graduate School, University of Science and Technology Beijing, Foshan 528399, China; 8 Department of Electrical and Electronic Engineering, The Hong Kong Polytechnic University, Hong Kong, China; changyuan.yu@polyu.edu.hk; 9 Yunsheng Intelligent Technology Co., Ltd., Tianjin 300457, China; cfp@ikingtec.com; 10 Shanxi Information Industry Technology Research Institute Co., Ltd., Taiyuan 030012, China; shangguan_xk@163.com

**Keywords:** IIoT, semantic communication, slot attention, industrial image transmission

## Abstract

In the Industrial Internet of Things (IIoT), vision-based industrial detection technology is crucial in the production process and can be used in many smart manufacturing applications, such as automated production control and Non-Destructive Evaluation (NDE). To enable timely and accurate decision-making, the network must transmit product status information to the server under stringent requirements of ultra-reliability and low latency. However, traditional pixel-centric industrial image transmission consumes additional bandwidth, and existing deep learning-based semantic communication systems rely on costly manual annotations. To overcome these limitations, this paper proposes a novel object-centric semantic communication framework based on improved slot attention for Multiple-Input Multiple-Output (MIMO) transmission in a 6G smart manufacturing scenario. First, we propose an improved slot attention method based on unsupervised learning for real-world manufacturing image datasets. The proposed method decouples complex industrial images into different object instances, each corresponding to an independent semantic component slot, effectively isolating task-related visual targets from redundant backgrounds. Furthermore, we propose a priority-based semantic transmission strategy. By quantifying the task-relevant importance of each semantic slot and jointly matching MIMO sub-channels, our method optimizes industrial image transmission streams, ensuring the reliable transmission of the important semantic information. Extensive simulation results demonstrate that the proposed framework significantly enhances communication transmission efficiency. Even under constrained bandwidth ratios and a low Signal-to-Noise Ratio (SNR), our framework achieves superior visual reconstruction quality and improves the Peak Signal-to-Noise Ratio (PSNR) by 4.25 dB compared to existing benchmarks.

## 1. Introduction

In the context of Industry 4.0 and the emerging Industry 5.0, traditional Non-Destructive Testing/Evaluation (NDT/NDE) technology [1] is being integrated with enabling technologies such as the Industrial Internet of Things (IIoT) and artificial intelligence (AI), giving rise to the concept of NDE 4.0 [2,3], which is driving a profound paradigm shift in modern manufacturing. In particular, by integrating physical machinery with digital networks, smart factories can achieve autonomous control, real-time monitoring, and predictive maintenance [4]. However, as the scale of the IIoT expands and the adoption of NDE 4.0 increases, the generation of data grows exponentially, imposing higher requirements on wireless networks, requiring massive data throughput, Ultra-Reliable and Low-Latency Communications (URLLCs), and high-precision computing and positioning capabilities [5]. To address these demands, the 5th Generation (5G) mobile communication system first considers massive Machine-Type Communication (mMTC) that includes industry scenarios in cellular networks [6]. The concept is further developed in the 6th Generation (6G) mobile communication system. 6G systems consider integrated artificial intelligence, massive Multi-Input Multi-Output (MIMO) communication, and semantic communication to better support automation and orchestration in industrial scenarios [7].

Conventional communication systems aim at accurately reconstructing the original data bit by bit. However, as physical layer technology gradually approaches the Shannon limit, relying on spread spectrum and higher-order modulation is no longer sufficient to meet the exponential growth in data demand for the IIoT. Unlike traditional systems that focus on the accuracy of transmitted symbols, semantic communication systems focus on the meaning conveyed by symbols [8,9]. By employing Joint Source and Channel Coding (JSCC) based on deep learning (DL), semantic communication filters out task-irrelevant data and transmits only the core semantic features [10]. This goal-oriented communication approach can significantly reduce communication overhead, minimize latency, and maintain robustness, which is highly suitable for future 6G smart factories [11,12]. Moreover, in 6G smart factories, semantic communication is suitable for goal-oriented tasks, which reduces redundant and irrelevant data, thus greatly improving production efficiency [13].

While semantic communication has remarkable success in the text [14] and video domains [15], research on its application in industrial image transmission remains limited. The current semantic communication systems process industrial images by operating on low-level visual units, such as pixels or image patches [10]. For example, Convolutional Neural Networks (CNNs) extract features from local pixel grids [16], and vision transformers (ViTs) model global dependencies among a sequence of pixel-patches [17]. However, most industrial images are composed of static and irrelevant backgrounds, with crucial semantic information concentrated on a few target products. If images are recognized as a collection of pixels, the channel encoder would consume valuable channel bandwidth and transmit power for a large number of meaningless background pixels, wasting limited wireless communication resources and impacting the performance of downstream tasks. On the other hand, semantic communication systems that rely on supervised learning require large amounts of accurate labels for effective training [18,19]. However, in practical factory scenarios, obtaining labeled data is often difficult due to the dynamic and complex nature of the industrial environment and data privacy.

To overcome these limitations, object-centric representation learning (OCL) has the potential to improve sample efficiency and model robustness [20]. A representative work in OCL is slot attention, which utilizes an iterative attention mechanism to convert an input image into a set of object representations [21]. Unlike traditional object detection architectures, slot attention achieves object-level decoupling through unsupervised learning, eliminating the need for manually labeled real labels. By extracting highly compact and task-related semantic information through slot attention, the transmitter of the semantic communication system does not need to transmit the original and complete image. The advantages of slot attention are well-suited for industrial tasks. Existing studies have demonstrated its effectiveness on simple datasets, where low-level features indicate pixel allocation. However, existing methods require further study to handle complex real-world data.

Motivated by the demand for transmitting information of multiple objects in harsh industrial environments, existing studies have investigated JSCC, including Single-Input Single-Output (SISO)-oriented JSCC frameworks and extensions to MIMO systems [22]. However, in complex industrial scenarios, minor information loss caused by the harsh transmission environment can cause severe semantic distortion, making it difficult to recover critical industrial content. This paper addresses the gap between existing pixel-centric image transmission frameworks and the requirements of object-oriented tasks in the IIoT. Specifically, we employ an improved slot attention method to extract object-centric semantic representations from industrial images. Furthermore, leveraging the better spatial degrees of freedom of MIMO transmission compared to SISO, we propose a priority-based semantic transmission strategy that evaluates the importance of each slot for the task and matches high-priority slots to better MIMO sub-channels, while assigning less important slots, such as background and other environmental information, to the low-response MIMO sub-channels. In this way, we achieve differential protection of task-related semantic information, realizing a task-relevant semantic communication system for MIMO transmission in industrial scenarios. The major contributions of the paper are summarized as follows:

- We construct an industrial image dataset for a smart factory scenario and propose an improved slot attention method based on unsupervised learning. This method can extract different object instances from complex real-world images and assign them to independent slots. This achieves high-precision, unlabeled semantic segmentation, saving the heavy manual annotation costs required for industrial image transmission.
- We design a new semantic communication framework that integrates the improved slot attention method with MIMO transmission. By transmitting highly compressed, object-centric semantic slots instead of raw pixels, our task-oriented system alleviates the burden on wireless networks and boosts transmission efficiency in complex industrial applications.
- We propose a novel priority-based semantic transmission strategy to realize differential protection of task-related semantic information. Evaluating the task-related importance of each slot and matching it with the channel state of the MIMO sub-channels ensures that the most critical semantic information is prioritized, reducing redundant information transmission and thus improving communication efficiency.
- Finally, we conduct comprehensive experiments on our industrial image dataset to validate the effectiveness of the proposed system. Extensive simulation results demonstrate that our object-centric semantic communication system outperforms existing benchmarks, maintaining a high Peak Signal-to-Noise Ratio (PSNR) and robust visual reconstruction quality, even under constrained bandwidth and low Signal-to-Noise Ratio (SNR) regimes.

## 2. Related Work

Under the NDE 4.0 paradigm, modern inspection workflows have integrated with digital connectivity, AI, and industrial infrastructures [1], leveraging neural networks, vision transformers, or hybrid learning models to automate defect detection, anomaly recognition, and quality assessment [23]. Industrial detection is evolving from manual observation and pixel-level analysis to data-driven, task-oriented semantic understanding. However, the robust transmission of high-resolution data over bandwidth-constrained wireless links is a major bottleneck for practical application in 6G industrial factories. Traditional methods of visual information transmission and processing usually regard images as a collection of pixels or local patches, ignoring the natural combination structure of objects in the visual scene. This pixel-based representation has significant limitations in industrial manufacturing contexts, where actual tasks often focus only on a small set of key objects related to production, inspection, or control. The transmission of large amounts of background pixels or redundant details not only increases the communication load but also complicates the system. Therefore, extracting more structured and semantically meaningful representations at the perceptual level and transmitting them efficiently and robustly has become an important research direction in intelligent industrial communications [24]. To address this challenge, semantic communication, as an emerging communication paradigm, provides an effective solution.

Unlike traditional communication paradigms, which target bit-level error rates, semantic communication emphasizes preserving the “meaning” or task relevance of the information to the greatest extent under limited communication resources [14]. Deep learning-based semantic communication systems significantly improve robustness under low-SNR and bandwidth-limited conditions by jointly modeling semantic encoding, channel effects, and semantic decoding end-to-end [25]. Research in this field has expanded from text-based semantic communication to Internet of Things (IoT) and edge intelligence scenarios, with a focus on model lightweighting, distributed deployment, and semantic preservation performance [26]. Semantic communication has significant application potential in future intelligent wireless networks and smart factories, especially in scenarios that require high real-time performance and reliability [27].

To achieve efficient semantic transmission under complex channel conditions, advanced source-channel coding methods need to be introduced to ensure the effective transmission of task-relevant semantics in wireless communication. In complex and dynamic wireless channel conditions, Deep Joint Source-Channel Coding (DJSCC) has become a key technology for achieving efficient semantic transmission. The DJSCC method learns continuous latent spaces and directly maps them to channel inputs, avoiding the performance bottlenecks of traditional source-channel separation designs under conditions of short code lengths and channel mismatch [28]. Recent research has focused on improving the adaptability and engineering feasibility of DJSCC, including adaptive bandwidth and SNR designs [29], constellation constraints to ensure compatibility with digital communication systems [30], and extended models for MIMO channels. Additionally, some studies have incorporated semantic importance into joint coding objectives, enabling the system to prioritize protecting more critical features for the task, thus improving semantic transmission efficiency with limited communication resources [24,31]. However, these importance-aware DJSCC methods mainly operate on patch-level representations and do not decompose the visual scene into object-centric semantic representations.

With the success of Transformer in the visual domain, semantic communication based on vision transformer (ViT) models images as a series of semantic tokens and utilizes an attention mechanism to characterize the importance of different image regions. Existing studies have leveraged the attention weights of ViT or learned importance mappings to perform importance-aware quantization, non-uniform feature protection, and adaptive resource allocation [17,32]. Some works further incorporate Channel State Information (CSI) into the Transformer architecture, enabling semantic feature mappings to dynamically adjust according to channel conditions, thereby enhancing system robustness and transmission efficiency in time-varying wireless environments [33]. However, these methods still primarily operate at the patch or region level and have not explicitly introduced object-level semantic structures.

In recent years, object-centric representation learning has provided a new paradigm for visual scene modeling. These methods introduce object-level latent variables to decompose complex scenes into interchangeable and composable object representations. Slot attention [21], as a representative method, aggregates perceptual features into a set of finite object slots through an iterative attention mechanism, enabling unsupervised object discovery and binding. Subsequent research has extended this idea to video scenes, incorporating temporal consistency to model dynamic object changes and demonstrating the stability and scalability of object slots in complex real-world environments [34,35]. Meanwhile, Transformer-based object dynamics modeling methods have further shown that object-level representations not only offer good interpretability but can also serve as effective carriers of high-level semantic information, supporting inference, prediction, and downstream tasks [36]. Recent studies have shown that combining strong self-supervised visual features with slot attention-based object decomposition can improve object-centric learning performance. However, these studies are mainly intended for visual representation learning, object discovery, or scene understanding, rather than for semantic communication over MIMO transmission systems.

In industrial manufacturing and industrial IoT scenarios, communication tasks typically have an object-oriented nature, such as component identification, defect detection [37], and assembly status verification. While previous studies have conceptually explored the value of semantic information processing in industrial systems, concrete methods that deeply integrate object-centric visual representations with channel-adaptive semantic communication remain scarce. Existing methods struggle to balance object-level semantic extraction, semantic importance evaluation, communication resource allocation, and robustness under complex channel conditions [32,38].

Based on the current research, this paper proposes an object-centric semantic communication framework for industrial manufacturing scenarios. Different from existing importance-aware DJSCC and ViT-based semantic communication frameworks, which prioritize patch-level or feature-level representations, the proposed framework uses improved slot attention to extract object-level semantic representations. In addition, unlike previous object-centric learning studies that focus on visual understanding, our framework integrates object-centric learning into a DJSCC-based MIMO communication system, realizing priority-based semantic transmission. The proposed framework extends semantic communication to object-centric wireless semantic transmission in industrial scenarios.

*Notation*: In this paper, boldface letters, such as A and a, indicate matrices or vectors. $C^{M\times N}$ denotes a complex-valued matrix of size M×N. $A^{T}$, $A^{H}$, $A^{-1}$, and $A^{†}$ denote the transpose, conjugate transpose, inverse, and pseudo-inverse of matrix A, respectively.

## 3. System Model

We consider an MIMO system where both the transmitter and receiver are equipped with *M* antennas. The objective is to transmit a source image $S\in R^{h\times w\times 3}$ to the receiver, where *h*, *w*, and 3 represent the height, width, and RGB color channels of the image, respectively. The image is encoded into a channel input matrix $X\in C^{M\times k}$, where *k* denotes the number of channel uses allocated to transmit one image. We define the bandwidth ratio as R≜k/n, which denotes the number of channel symbols used per source dimension, with n=3hw denoting the number of source symbols. The MIMO channel is modeled as a block-fading channel, where the channel matrix H remains constant within the transmission of a single image block and varies independently across blocks. The MIMO channel model can be written as

(1) Y=HX+W,

where $Y\in C^{M\times k}$, $H\in C^{M\times M}$, and $X\in C^{M\times k}$ denote the received signal matrix, channel gain matrix, and transmitted signal matrix, respectively. The entries of H as $H∼\mathrm{CN}(0,\sigma_{h}^{2})$ follow a complex Gaussian distribution with zero mean and variance $\sigma_{h}^{2}$. Additionally, $W\in C^{M\times k}$ represents the Additive White Gaussian Noise (AWGN) term, whose entries $W∼\mathrm{CN}(0,\sigma_{w}^{2})$ also follow a Gaussian distribution with zero mean and variance $\sigma_{w}^{2}$.

Given the received signal matrix Y, the receiver reconstructs the source image as $\hat{S}\in R^{h\times w\times 3}$. The reconstruction quality is quantified through the Peak Signal-to-Noise Ratio (PSNR). The PSNR metric serves as an indicator of image distortion at the per-pixel level, defined as

(2) $$\mathrm{PSNR}≜10\log_{10}\left(\frac{{∥S∥}_{\infty }^{2}}{\mathrm{MSE}(S,\hat{S})}\right)\;\left(\mathrm{dB}\right)$$

where $\mathrm{MSE}\left(S,\hat{S}\right)≜\frac{1}{3hw}{∥S-\hat{S}∥}_{2}^{2}$ is the mean squared error (MSE) between S and $\hat{S}$. Actually, there are two transmission schemes: open-loop MIMO and closed-loop MIMO. In an open-loop MIMO system, CSI is only available at the receiver, enabling it to equalize the transmitted signals and subsequently decode the image. For the closed-loop MIMO system, the CSI is accessible to both the transmitter and receiver, which allows them to apply precoding and power allocation at the transmitter and MIMO equalization at the receiver, thereby improving the image transmission quality. For simplicity, we only consider the closed-loop MIMO system in this paper, and open-loop MIMO systems will be discussed in future work.

For the semantic communication system, source and channel coding are jointly optimized. We exploit deep learning (DL) technologies to parameterize the encoder and decoder functions, which are trained jointly on an image dataset and the channel model. We denote the DJSCC encoder and decoder by $f_{\theta }$ and $f_{\phi }$, respectively, where θ and ϕ denote the network parameters. We have

(3) $$X=f_{\theta }\left(S,H,\sigma_{w}^{2}\right).$$

Unlike the traditional separate source-channel coding scheme, the transmitter does away with power allocation. Instead, we leverage the DJSCC encoder to perform feature extraction and channel symbol mapping, and power allocation, all at once. Intuitively, the DNN is expected to transmit critical features over sub-channels with higher SNRs, thereby improving the transmission performance.

We note that one option is to train the encoder and decoder networks directly, hoping they will learn to exploit the spatial degrees of freedom that the MIMO channel provides. We will instead follow the model-driven approach, where we will exploit the Singular-Value Decomposition (SVD) and first convert the MIMO channel into sub-channels. The received signal can be written as

(4) Y=HVX+W=UΣX+W,

where $U\in C^{M\times M}$ and $V\in C^{M\times M}$ are unitary matrices. To simplify training, we apply MIMO equalization at the receiver by multiplying $\Sigma^{†}U^{H}$ to obtain the following:

(5) $$\hat{X}=\Sigma^{†}U^{H}Y=X+\hat{W},$$

where $\hat{W}≜\Sigma^{†}U^{H}W\in C^{M\times k}$ is the equivalent noise term. Finally, we feed both $\hat{X}$ and the CSI into the DJSCC decoder to recover the image as

(6) $$\hat{S}=f_{\phi }\left(\hat{X},H,\sigma_{w}^{2}\right).$$

Our scheme integrates DJSCC with algorithms from a previous model-driven MIMO solution, including SVD-based precoding and equalization. The objective is to improve the performance of the DJSCC approach by exploiting the conventional MIMO design.

## 4. Proposed Method

In this section, we present a novel semantic communication system using the DJSCC framework, aiming to improve the efficiency of industrial image transmission in MIMO systems. Specifically, we first discuss the overall design of the proposed system, then improve the ability of slot attention on real-world datasets by integrating the DINO module. Next, we propose a priority-based semantic transmission strategy, which achieves efficient transmission by matching the task-related importance of each slot with the MIMO channel quality. Following this, we discuss the design of the encoder and decoder for the DJSCC scheme. Finally, we explain how our loss function is defined.

### 4.1. System Overview

**Figure 1:**
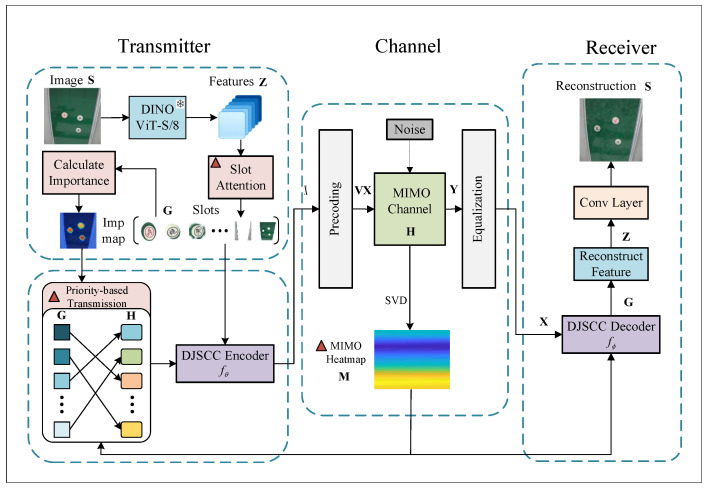
The pipeline of the semantic communication system using the DJSCC framework.

The pipeline of the proposed semantic communication system is depicted in Figure 1. Specifically, the input image S is fed into the DINO module to extract semantic features Z. Then, the slot attention method is used to decompose Z into object-centric representations G, and we calculate the importance of each slot. On the channel side, SVD is performed on the MIMO channel to obtain a channel heatmap, then the MIMO sub-channels are matched with slots to realize a priority-based semantic transmission strategy. Next, G is encoded by the DJSCC encoder and represented as X. X is precoded as VX and transmitted through the MIMO channel. At the receiver, the received signal Y is processed by channel equalization to obtain $\hat{X}$, and then the DJSCC decoder reconstructs it into feature $\hat{G}$. Finally, the feature $\hat{G}$ is mapped to an RGB image to obtain the reconstructed image $\hat{S}$. In a closed-loop MIMO system, CSI is fed to the encoder and decoder and facilitates the DJSCC encoding and decoding process in the form of heatmaps.

### 4.2. Improved Slot Attention Method

Powerful feature extraction abilities have proven helpful in extending object-centric learning methods to complex real-world datasets [39]. The foundation of our method is the extraction of rich semantic features using a frozen DINO vision transformer. Compared with conventional CNN or supervised ViT backbones that require expensive label annotation, self-supervised DINO provides rich semantic information without using task-specific labels, which makes it suitable for object decomposition in industrial scenarios. Given an input image $S\in R^{h\times w\times 3}$, the DINO outputs patch tokens, denoted as

(7) $$Z=\Phi_{\mathrm{DINO}}\left(S\right)\in R^{N\times D_{\mathrm{feat}}},$$

where *N* is the sequence length of the patches and $D_{\mathrm{feat}}$ is the feature dimension. We adopt original slot attention, which includes GRU and residual Multi-Layer Perceptron (MLP) modules as in [21] to turn the set of input features Z into a set of *T* slot vectors $B\in R^{T\times D_{\mathrm{slot}}}$, where $D_{\mathrm{slot}}$ denotes the slot feature dimension. The slots are randomly initialized and iteratively compete for input features through an attention mechanism. Following the ViT design [40], we add positional encoding to the input features to enhance the model’s ability to distinguish objects that are semantically similar but spatially separated. The attention coefficients are calculated as

(8) $$A_{t,n}=\frac{\exp\left(\frac{q_{t}\cdot k_{n}}{\sqrt{D_{\mathrm{slot}}}\cdot \tau }\right)}{\sum_{n^{\prime }=1}^{N}\exp\left(\frac{q_{t}\cdot k_{n^{\prime }}}{\sqrt{D_{\mathrm{slot}}}\cdot \tau }\right)},$$

where $q_{t}$ and $k_{n}$ are query and key vectors, *t* is the index of slots and *n* is the index of input patch, and τ is the temperature parameter, which intensifies slot competition and encourages more slot assignments. We use an MLP decoder, similar to a spatial broadcast decoder, applied independently to each slot. The MLP decoder reconstructs features by taking a weighted sum across the slots and produces an alpha map $\alpha_{t}$, which represents the corresponding activated *t*-th slot.

In industrial scenarios, the semantic information contained in different objects relates to different downstream tasks. To leverage the spatial degrees of freedom of MIMO communication and improve industrial production efficiency, we need to prioritize the transmission of important semantic information on channels with large channel capacity. Inspired by previous importance-aware semantic communication methods [17], we define an importance metric $I_{t}$ for each slot, but our metric evaluates importance at the object-level and is quantified by three complementary factors derived from slot attention outputs. This metric is motivated by the observation that important objects in images are typically spatially concentrated, compact, and semantically informative. The importance metric includes: (i) Concentration ($C_{t}$): The ratio of the peak to the mean attention weight, measuring spatial focus. High concentration indicates a slot represents a compact object rather than a broad background. (ii) Feature Richness ($R_{t}$): The $\ell_{2}$-norm of slot representation, measuring richness of semantic information. (iii) Compactness ($Q_{t}$): The inverse of attention entropy, measuring spatial coherence. These factors are normalized to [0, 1] and combined with learned weights, then the importance score for each slot is formulated as

(9) $$I_{t}=\omega_{c}{\tilde{C}}_{t}+\omega_{r}{\tilde{R}}_{t}+\omega_{p}{\tilde{Q}}_{t},$$

where ${\tilde{C}}_{t}$, ${\tilde{R}}_{t}$, and ${\tilde{Q}}_{t}$ denote normalized values. Subsequently, these slot importance scores are projected onto the feature patches via the attention map, constructing a semantic importance map $I_{\mathrm{map}}\in R^{h\times w}$, which will be used in the priority-based semantic transmission strategy.

### 4.3. Priority-Based Semantic Transmission Strategy

To fully utilize the spatial degrees of freedom in MIMO systems, we propose a priority-based semantic transmission strategy that prioritizes the allocation of important semantic information to the sub-channel with the lowest equivalent noise for transmission. First, to obtain the equivalent additive noise intensity of the MIMO sub-channels, we construct a channel heatmap from CSI. We define $P_{m}\in R^{M\times k}$ as the average power of the equivalent additive noise term $\hat{W}$. In the closed-loop MIMO system, $P_{m}\in R^{M\times k}$ is defined as

(10) $$P_{m}=\sigma_{w}^{2}\Sigma^{†}U^{H}J_{M\times k},$$

where J is a matrix of ones. To ensure the encoder can be trained effectively, we concatenate two matrices $\frac{1}{2}P_{m}$ to get the shape of $R^{M\times 2k}$, and reshape it into $M\in R^{N\times \frac{MN}{2k}}$, which represents the equivalent noise term faced by each real encoder output element. The channel heatmap M is constructed as

(11) $$M=\mathrm{reshape}\left(\mathrm{concat}\left(\frac{1}{2}P_{m},\frac{1}{2}P_{m}\right)\right),$$

where concat(·) and reshape(·) denote the concatenation and reshape operations, respectively. By embedding this heatmap into the DJSCC framework, the system transforms CSI into a spatially structured bias, enabling the model to be used in the system with varying numbers of antennas.

Finally, we need to pair semantic slots with MIMO sub-channels optimally. We sort the feature tokens extracted by the DINO module in descending order according to the semantic importance map $I_{\mathrm{map}}$ and divide them into $\left\{G_{1},G_{2},\ldots ,G_{M}\right\}$, where m∈{1,…,M} is the index of MIMO transmit antennas. Each group $G_{m}$ contains a subset of feature tokens with similar task relevance. Meanwhile, the parallel MIMO sub-channels are sorted by their singular values $\left\{\sigma_{1},\sigma_{2},\ldots ,\sigma_{M}\right\}$. We employ a greedy matching algorithm to allocate the $G_{m}$ with the highest semantic priority to the sub-channel with the highest singular value, i.e., the channel with the lowest equivalent noise. By doing so, we can obtain the index of the mapping relationship. This mapping relationship between semantic importance and channel capacity ensures that task-related semantic information is protected by the robust MIMO channel in industrial scenarios, while redundant background information is allocated to sub-channels with high equivalent noise. In SISO transmission, all semantic information experiences the same channel condition because only a single transmission stream is available. By contrast, MIMO provides multiple sub-channels with different qualities, making differentiated protection of semantic information possible and thereby improving the efficiency of industrial image transmission.

Since MIMO sub-channels are naturally sorted by their singular values and semantic feature groups are also sorted by their semantic importance, the greedy matching algorithm is sufficient for the current priority-based semantic transmission strategy. Additionally, compared with the Hungarian algorithm, which incurs higher computational complexity, the computational complexity of the greedy matching algorithm is lower. Learning-based allocation strategies are more beneficial when the assignment objective is less explicit or the channel environment becomes highly dynamic. This also motivates one of our future studies to investigate adaptive slot discovery and learning-based MIMO allocation strategies for more complex time-varying scenarios and dynamic channel states.

### 4.4. DJSCC Encoder and Decoder

Unlike existing frameworks using Transformer architecture [41], we employ an efficient MLP-based encoder and decoder architecture in the DJSCC framework. Because the slot attention method effectively captures the object-centric semantic context, the simplified architecture saves on the computational and memory overhead required for the attention mechanism, making the model more suitable for deployment in industrial edge networks.

For the feature group $G_{m}$ allocated to the *m*-th sub-channel, we use an MLP-based encoder $f_{\theta }$ to achieve adaptive bandwidth compression, which compresses the high-dimensional features into a compact channel symbol matrix. To enable the DJSCC encoder to have physical channel awareness, we scale the encoded symbols using the average noise power $P_{m}$ from heatmap M. Then, the transmit symbols for the *m*-th feature group are formulated as

(12) $$X_{m}=f_{\theta }\left(G_{m},P_{m}\right),$$

where $P_{m}$ guides the encoder’s power allocation during MIMO transmission, allowing the encoder to allocate more transmission power to high-priority semantic information.

At the receiver, after the MIMO channel equalization process as detailed in (5), the received symbol ${\hat{X}}_{m}$ can be obtained for each sub-channel. A symmetric MLP-based decoder $f_{\phi }$ is used to reconstruct semantic features. To compensate for channel loss, the average noise power $P_{m}$ is concatenated with the received symbols as a conditional prior. The reconstructed feature group ${\hat{G}}_{m}$ is given as

(13) $${\hat{G}}_{m}=f_{\phi }\left([{\hat{X}}_{m},P_{m}]\right)\in R^{N\times D_{\mathrm{feat}}}.$$

After decoding the *M* feature groups from each sub-channel, they must be spatially realigned. Using the mapping relationship index from the transmitter, each feature can be restored to its original spatial coordinates. The reconstructed features are $\hat{Z}\in R^{N\times D_{\mathrm{feat}}}$. Finally, to reconstruct the RGB image from the feature matrix, we employ a progressively transposed convolutional network to decode the feature matrix $\hat{Z}$ into the reconstructed industrial image $\hat{S}\in R^{h\times w\times 3}$. In both image processing and MIMO transmission, we operate based on feature tokens. By processing feature tokens in parallel, we can improve the transmission efficiency of massive amounts of industrial images.

### 4.5. Loss Function

During the training phase of the model, we adopt a two-stage strategy. In the first training phase, we primarily train the improved slot attention module to ensure the model has good segmentation capabilities for objects in industrial images. Then, given *T* slots $B\in R^{T\times D_{\mathrm{slot}}}$, the self-supervised features are reconstructed as ${\hat{Z}}_{\mathrm{SA}}\in R^{N\times D_{\mathrm{feat}}}$. The loss function is

(14) $$L_{1}={∥Z-{\hat{Z}}_{\mathrm{SA}}∥}^{2},$$

where Z is the original DINO feature. The goal is to encourage slots to capture object-centric semantic features in real-world images, rather than just low-dimensional pixel features.

In the second training phase, the DJSCC encoder–decoder undergoes end-to-end training on a simulated MIMO channel. To ensure semantic integrity after MIMO transmissions, the loss function is defined as

(15) $$L_{2}=\mathrm{MSE}\left(S,\hat{S}\right).$$

We train the model to search for the optimal parameters θ and ϕ of the encoder and decoder by minimizing $L_{2}$ as $\left(\theta ,\phi \right)=\arg\min_{\theta ,\phi }E_{D}\left[L_{2}\left(\theta ,\phi \right)\right]$, where the expectation is obtained through the image and channel datasets.

## 5. Experimental Results

In this section, we conduct experiments to evaluate the performance of the proposed semantic communication framework. We demonstrate the superiority of the proposed framework by training on industrial image data and testing the image reconstruction quality and quantitative metrics.

### 5.1. Experimental Setup

To begin, we introduce the industrial image dataset we created ourselves [42], named the Chess dataset. We constructed a multi-crane visual sorting system by controlling the dynamic parameters of a conveyor belt and cranes, achieving the controllability of material sorting. We used deployed cameras to capture images of the material sorting process. The dataset has 250 images with dimensions 3 × 1920 × 1080 (RGB color, height, and width); each image contains 1∼3 chess pieces that need to be sorted, and we use 200 images as the training dataset. In the preprocessing stage, the original image size is too large, which is not easy for training. Then, we crop the image to a 512 × 512 pixel size from the center and perform data augmentation through random cropping.

Our model was implemented in PyTorch 2.8.0 with one GTX H800 GPU. We use a learning rate of $5\times 10^{-5}$, a 2k step linear warm-up followed by a linear decay, and a batch size of 16 with an Adam optimizer of $\beta_{1}=0.9$ and $\beta_{2}=0.999$ to train. In the first training phase, we employ DINO ViT/B-8 as a frozen feature extractor [43] and train the slot attention module for 2000 epochs. For the training parameters of the slot attention model, we set the number of slots K=5, the number of iterations to 3, the hidden layer dimension to 256, and the temperature τ=0.75. In the second phase, we train the DJSCC module of the semantic communication system. We consider a 5 × 5 closed-loop MIMO system, and the hidden layer dimension of the symmetric MLP-based encoder and decoder is 1024.

### 5.2. Performance Comparison

In this section, we comprehensively evaluate the performance of the proposed object-centric semantic communication framework by demonstrating the image reconstruction quality of the model on the test set. The PSNR is adopted as the main reconstruction metric, since it is a classical and widely used measure in wireless image transmission and DeepJSCC studies [44]. Additionally, we conduct comparative experiments under different SNR values, different bandwidth ratios, and different CSI estimation errors, proving that the proposed framework has good robustness to communication systems under different conditions. Finally, we perform ablation experiments to compare to the traditional framework.

#### 5.2.1. General Performance

At first, we analyze the visual reconstruction quality of the transmitted industrial images. We train the model under the experimental setup, where the training SNR=10 and bandwidth ratio R=1/12.

**Figure 2:**
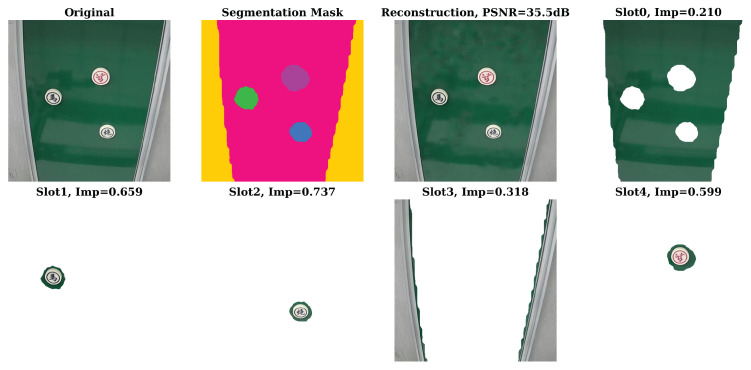
The quality of the image reconstruction and the object separation performance of the semantic communication framework based on the improved slot attention method.

In Figure 2, we demonstrate the quality of the image reconstruction after MIMO transmission and the object separation performance of the slot attention module on the image dataset. Our proposed framework achieves exceptional semantic fidelity for the task-relevant objects. As shown in the reconstruction result, our proposed framework achieves superior semantic fidelity for task-related objects. This is a benefit of the improved slot attention method and priority-based semantic transmission strategy, which allows the system to prioritize the allocation of MIMO sub-channels with better channel conditions to object slots, reducing or even discarding redundant background details. Moreover, by introducing the designed slot importance calculation matrix, it effectively distinguishes the importance of objects from the background, which also contributes to realizing an efficient semantic transmission strategy.

It is noted that the semantic importance metrics may vary depending on the specific downstream tasks. For the same material sorting task, simply adjusting the number of slots *T* in the model is sufficient. But for tasks like subtle defect detection, the weight of feature richness needs to be increased to capture subtle texture variations. Similarly, for the assembly verification task, enhancing the spatial consistency and compactness metrics is helpful. Moreover, to make the model applicable to many other industrial tasks, it is necessary to adjust the attention weights in the model and the task-specific semantic importance calculation mechanism. By fine-tuning the model parameters, the proposed model can be applied to subtle defect detection, assembly verification, object monitoring, and other industrial tasks. To enable the model to handle multiple industrial tasks simultaneously, we view the design of a superior method to distinguish the semantic importance of objects across multiple tasks to support the applicability of the model in multiple downstream tasks as future work.

**Figure 3:**
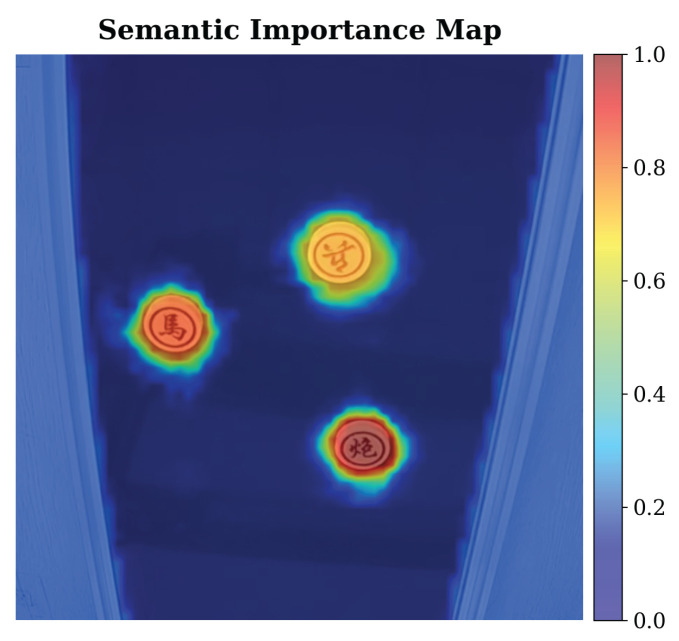
A semantic importance map of the Chess dataset.

To demonstrate the effectiveness of the proposed method in calculating the importance matrix for each slot, we provide the semantic importance map $I_{m}$ as shown in Figure 3. The result shows that the three chess pieces in the image are presented in warm colors due to higher semantic importance, while other objects with low semantic importance, such as backgrounds and environments, are presented in cool colors. Object instances in an image are typically represented in a highly semantic, concentrated, and compact form. Therefore, the proposed method has a certain applicability for task-oriented semantic communication systems, which can reduce the transmission of redundant background information and improve the transmission efficiency of task-related semantic information.

**Figure 4:**
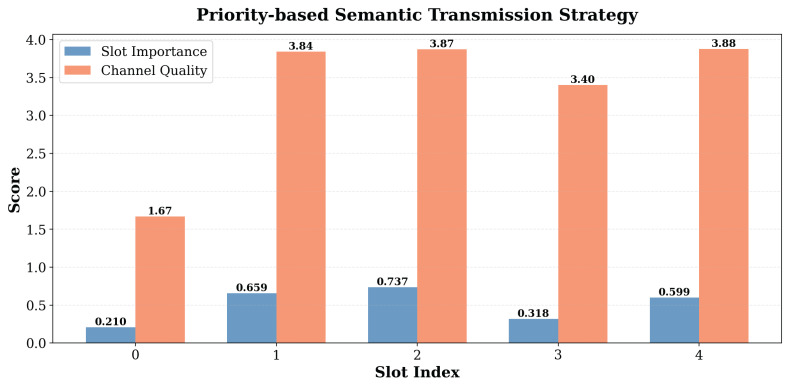
Importance mapping relationships using the priority-based semantic transmission strategy.

Furthermore, Figure 4 illustrates the proposed priority-based semantic transmission strategy, which realizes the matching relationship between slots and MIMO sub-channels. The orange bars labeled “Channel Quality” represent the values obtained after SVD decomposition on the MIMO channel. A higher value indicates better channel conditions with lower noise power for that sub-channel. On the other hand, the blue bars labeled “Slot Importance” represent the importance score obtained using the proposed semantic importance calculation method. The result shows a good matching relationship between slots and the MIMO channels under the experimental setup. Even for more complex images, we can discard redundant background information and prioritize the transmission of task-related semantic information, effectively improving the efficiency of industrial image transmission.

#### 5.2.2. Different Bandwidth Ratio Performances

**Figure 5:**
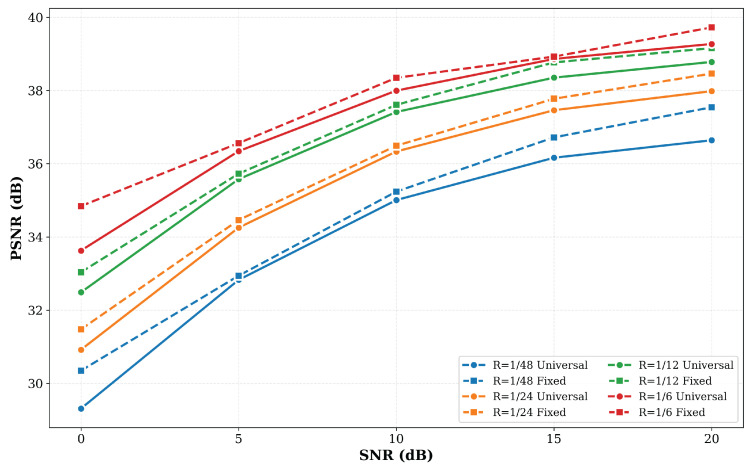
Performance comparisons of the model trained under fixed SNR and universal SNR strategies at different SNRs and bandwidth ratios *R*.

We evaluate the semantic communication model under the setup where the training SNR is the same as the testing SNR, denoted by ${\mathrm{SNR}}_{\mathrm{test}}$. We set ${\mathrm{SNR}}_{\mathrm{test}}\in \left\{0,\;5,\;10,\;15,\;20\right\}$ dB and the bandwidth ratio R∈{1/48, 1/24, 1/12, 1/6}; this is the fixed SNR strategy. In addition, we consider a random SNR training strategy to evaluate the SNR adaptability of the model. Specifically, we train the model at random SNR values uniformly sampled from [0,20] dB and test it at ${\mathrm{SNR}}_{\mathrm{test}}\in \left\{0,\;5,\;10,\;15,\;20\right\}$, denoted as the universal SNR strategy. As shown in Figure 5, when the ${\mathrm{SNR}}_{\mathrm{test}}$ increases, a higher PSNR value can be obtained, indicating the model achieves better image reconstruction quality under better channel conditions. More importantly, for models with different bandwidth ratios *R* under the same ${\mathrm{SNR}}_{\mathrm{test}}$, the model trained with a universal SNR strategy shows a slight performance degradation compared to the model trained with a fixed SNR strategy, but the degradation does not exceed 2 dB. Thus, this degradation is acceptable, demonstrating that our model has good SNR adaptability to different channel environments and can achieve a good PSNR value even in relatively harsh channel environments, which is crucial for its transfer to various industrial applications.

#### 5.2.3. Robustness to CSI Estimation Errors

**Figure 6:**
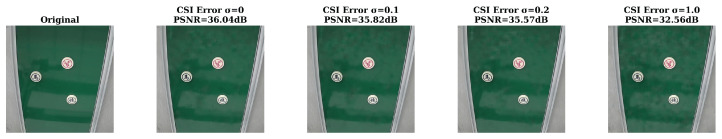
Visual comparisons of image reconstruction in the presence of CSI estimation errors, where the model is trained with a universal SNR strategy and validated at ${\mathrm{SNR}}_{\mathrm{test}}=10$ and R=1/12.

To evaluate the robustness of MIMO systems to channel estimation errors, we assume that H is imperfectly estimated at the receiver as $\hat{H}$. As [45], we assume $E_{n}≜H-\hat{H}$, where the entries of $E_{n}$ are zero-mean complex Gaussian with variance $\sigma_{e}^{2}$. First, we train the model under the universal SNR strategy and validate it at ${\mathrm{SNR}}_{\mathrm{test}}=10$ and R=1/12. Then, we test the model at different $\sigma_{e}^{2}\in \left\{0,\;0.1,\;0.2,\;1.0\right\}$ values. Therefore, we consider imperfect $\hat{H}$ in both training and testing. The results are shown in Figure 6: the performance degrades as the CSI error increases. However, our proposed semantic communication framework remains effective. Although performance inevitably degrades, the quality of the target object in the reconstructed image remains clear, demonstrating that our model has good robustness to CSI estimation errors.

#### 5.2.4. Robustness to Different SNRs

**Figure 7:**
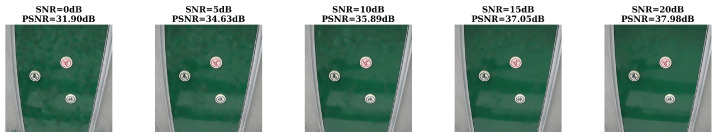
Visual comparisons of image reconstruction quality, where the model is trained with a universal SNR strategy and tested at ${\mathrm{SNR}}_{\mathrm{test}}\in \left\{0,\;5,\;10,\;15,\;20\right\}$ with R=1/12.

To further investigate the effect of channel SNR on model performance, Figure 7 shows the visualization comparisons of the image reconstruction using a model trained with the universal SNR strategy with R=1/12 and tested in ${\mathrm{SNR}}_{\mathrm{test}}\in \left\{0,\;5,\;10,\;15,\;20\right\}$, where the SNR and PSNR of the image are provided at the top of each visualization patch. It can be seen that the proposed system achieves good image reconstruction quality under different SNRs, which further demonstrates that our model has good SNR adaptability to different channel environments. Even at a low SNR, the background of the constructed image is largely blurred, but the chess pieces related to the material sorting task remain clearly visible. This demonstrates that we realize effective task-related semantic communication, where key semantic information is preferentially transmitted under superior MIMO sub-channels.

#### 5.2.5. Ablation Experiment

To evaluate the effectiveness of the proposed improved slot attention method and priority-based semantic transmission strategy, we conduct an ablation experiment on the model using the universal SNR strategy. The result of the ablation experiments is shown in Table 1. Specifically, when R=1/12, SNR=10 dB, the proposed model achieves PSNR improvements of 2.77 and 1.34 dB, respectively, compared to the model without slot attention and the model without channel heatmaps. Furthermore, compared to the baseline DJSCC model, the proposed model achieves PSNR improvements of 4.25 dB. Experimental results demonstrate that, under different SNRs and bandwidth ratios, our proposed method is helpful for the performance of industrial image transmission systems in closed-loop MIMO systems.

### 5.3. System Efficiency

To realize URLLC in 6G smart factories, evaluating the efficiency of the proposed system is crucial. In this section, we discuss the computational complexity, inference latency, and deployment feasibility to demonstrate that the proposed system can be deployed in resource-constrained industrial edge devices.

We compare the system efficiency between our proposed method and the DJSCC system based on the Transformer architecture. All comparisons are averaged over 50 runs with batch size 1, input resolution 224×224, M=2, SNR=10 dB, and bandwidth ratio R=1/24. The results are shown in Table 2. The results show that our proposed method has significantly fewer model parameters, about 72%, compared to the Transformer-based DJSCC system. Introducing a slot attention module reduces the parameter count of the DJSCC design; thus, MLP-based DJSCC avoids the $O(N^{2}D)$ complexity of the Transformer self-attention mechanism. Moreover, our system achieves an inference acceleration, reducing inference latency by 3.68 ms compared to the Transformer-based DJSCC system. However, it is inevitable that our system consumes more GPU memory, mainly due to the pre-trained DINO module, but our method can better handle industrial images from the real world and achieves task-related semantic communication. We will explore a lightweight semantic communication system for resource-constrained industrial edge networks as one of our future research directions.

On the other hand, the proposed semantic communication framework focuses on end-to-end semantic coding and transmission strategy design, without discussing the design for CSI estimation. The framework has similar channel requirements as conventional communication systems; the overhead for CSI estimation is approximated, with efficiency primarily determined by the transmission performance. However, the proposed semantic system achieves high overall efficiency. This is attributed to the proposed priority-based semantic transmission strategy, which significantly reduces the task-irrelevant background information requiring transmission. Furthermore, the industrial communication links are more structured compared to typical mobile communication environments, making CSI management more feasible. Future work will explore the design of open-loop MIMO systems and consider CSI feedback in multi-task communication models as in our previous work [46,47] for improved communication efficiency and performance of semantic communication.

## 6. Conclusions

In this paper, we propose a novel object-centric semantic communication framework for MIMO transmission in 6G industrial scenarios, aiming to support efficient visual information transmission in 6G smart factories and intelligent detection applications related to NDE 4.0. This framework breaks away from the traditional pixel-centric industrial image processing paradigm that relies on manual annotation, introducing an improved unsupervised slot attention method. This method decouples complex industrial images into different, task-related object instances, effectively avoiding the high cost of data annotation in industrial images while filtering out redundant background noise. Furthermore, we design a priority-based semantic transmission strategy that combines semantic importance with the physical characteristics of MIMO channels. By deterministically mapping task-related key slots to the optimal MIMO sub-channels, we achieve a highly robust semantic transmission strategy. Finally, we create our own image dataset from real-world industrial scenarios. Extensive evaluation on this dataset demonstrates that the proposed framework significantly improves industrial image transmission efficiency. Even under constrained bandwidth ratios and low SNRs, it still achieves a superior image reconstruction quality, and the PSNR is improved by 4.25 dB compared to the baseline method.

In future work, firstly, we will explore adaptive slot discovery and learning-based MIMO resource allocation algorithms to adapt to more complex time-varying scenarios and dynamic channel states. Secondly, we will investigate open-loop MIMO system designs and develop a lightweight DINO module alternative for task-oriented semantic communication systems, thereby promoting the deployment of edge nodes in next-generation industrial networks. Finally, considering that real-world industrial environments typically require the simultaneous execution of multiple tasks, we aim to extend our semantic communication framework into an end-to-end, multi-task-driven framework and evaluate it using richer metrics, such as the Structural Similarity Index Measure (SSIM), Learned Perceptual Image Patch Similarity (LPIPS), and task-oriented metrics.

## Figures and Tables

**Table 1:** Ablation experiment results.

Models	0 dB	5 dB	10 dB	15 dB	20 dB
**Proposal**	**32.16**	**35.58**	**37.78**	**38.95**	**39.04**
W/o slot	23.53	27.11	35.01	35.74	37.03
W/o heatmap	31.54	34.37	36.44	37.36	37.58
Baseline [27]	23.03	26.08	33.53	34.18	35.28

**Table 2:** System efficiency comparison.

Method	Params (M)	GPU Memory (MB)	Latency (ms)
**Proposed**	**29.99**	**614.8**	**9.42**
Transformer–DJSCC [27]	111.46	462.2	13.10

## Data Availability

The raw data supporting the conclusions of this article will be made available by the authors on request.
